# Clinical features of two Japanese siblings of neuronal ceroid lipofuscinosis type 1 (CLN1) complicated with TypeⅡ diabetes mellitus

**DOI:** 10.1016/j.ymgmr.2023.101019

**Published:** 2023-11-08

**Authors:** Kaoru Eto, Rina Itagaki, Ayumi Takamura, Yoshikatsu Eto, Satoru Nagata

**Affiliations:** a Tokyo Women's Medical University, Adachi Medical Center, Department of Pediatrics, Japan; b Tokyo Women's Medical University, Department of Pediatrics, Japan; c Tottori University Faculty of Medicine, Department of Pathobiological Science and Technology, Japan; d Advanced Clinical Research Center & Asian Lysosomal Research Center, Institute of Neurological Disorders, Japan

**Keywords:** CLN1, Lysosomal storage disorders, Deterioration, Blindness, Type II diabetes mellitus

## Abstract

Neuronal ceroid lipofuscinosis type1(CLN1), is a one form of the group of neuronal ceroid lipofuscinoses (NCLs), which is a neurodegenerative disorder characterized by progressive psychomotor deterioration, ataxia, epilepsy, and visual impairment. Neurological manifestations occur at a wide range of ages, from infancy to adulthood, but are most common in infancy. The prevalence of CLN1 is unclear; however, it is very rare in Japan and Europe. In Japan, only a few cases have been reported, two of infantile- and one of juvenile-onset type. Nonetheless, the clinical characteristics of Japanese patients and their relationship with the genotype have not been sufficiently investigated. Here, we report the cases of two siblings that presented with juvenile-onset (a 22-year-old man and a 29-year-old woman) CLN1 associated with type II diabetes mellitus. In both cases, visual impairment followed by learning disability was observed from school-age, and retinitis pigmentosa was noted on ophthalmological examination. These patients presented type II diabetes mellitus during their later teenage years. Brain magnetic resonance imaging (MRI) revealed marked atrophy of the cerebrum and cerebellum. The clinical symptoms lead to suspect NCLs. Decreased PPT1 enzyme activity in dried blood spot (DBS)and leukocytes were observed, and the genetic analysis revealed heterozygous missense variants in *PPT1*, c.550G > A/c.664 A > G (p. Glu184Lys/p. Lys216Glu). The latter variant of this patients was novel variant. The residual enzymatic activity of PPT1 in these cases is higher than that in the infantile type. CLN1 mutant cells are known to have altered subcellular expression and localization, enhanced lipid raft-mediated endocytosis, abnormal autophagy, and mitochondrial dysfunction. Although the prevalence of diabetes mellitus is high and the possibility of coincidental complications cannot be ruled out, we concluded that mitochondrial abnormalities are involved in insulin resistance and may be implicated in the development of type II diabetes mellitus. Further studies are needed to prove the correlation between CLN1 and diabetes mellitus.

## 1 Introduction

Neuronal ceroid lipofuscinoses (NCLs) are a heterogeneous group of neurodegenerative lysosomal storage disorders characterized by intracellular accumulation of auto fluorescent lipofuscin granules in the lysosomes of neurons, fibroblasts, and many other cells [1]. The clinical features of NCLs include progressive neurological deterioration with developmental delay, dementia, ataxia, epilepsy, and retinopathy. Traditionally, NCL has been classified into four types according to the age of onset: infantile, late infantile, juvenile, and adult. In addition to these classifications, several other variants have been reported from specific regions and races [2]. Subsequently, the pathogenesis of the disease was elucidated by biochemical and genetic tests, and the disease was classified into 13 types according to the clinical form (CLN1–14, CLN9 is absent): CLN1/PPT1, CLN2/TPP1, CLN5, CLN10/CTSD, CLN13/CTSF, and CLN11/GRN, which are characterized by lysosomal enzymes; and CLN3 and CLN7/MFSD8, which are known to have abnormal lysosomal membrane proteins. In addition, membrane proteins of the endoplasmic reticulum have been reported to be abnormal in CLN6 and CLN8, and proteins related to the periplasmic membrane have been reported in CLN4/DNAJC5 and CLN14/KCTD7, suggesting that a variety of mechanisms are involved in its pathogenesis [3,4]. The frequency of the disease types in Europe and the United States is as following: CLN3 (22.6%), CLN2 (21.2%), and CLN1 (19.6%) [5], and about 300 variants have been reported in these three disease types [6]. The exact frequency of this disease in Japan has not been fully evaluated.

CLN1 (MIM 256730) is caused by a deficiency in the lysosomal enzyme palmitoyl-protein thioesterase 1 (PPT1). The typical clinical presentation of CLN1 is onset in infancy (after 8 months of age) with microcephaly, epilepsy, psychomotor regression, and visual impairment, followed by being bedridden; most patients die around the age of 10 years. Electron microscopy images show granular osmophilic deposits (GRODs), most of which are Saposin A and Saposin D. Approximately 80 variants have been reported for CLN1, and most show different variants in different families. In Finland, the same missense variant (c.364 A > T; p. Arg122Trp) was found in most families. The majority of reported variants of CLN1 are severe, early onset infantile forms; however, variants with late-onset infantile, juvenile, and adult phenotypes have also been reported [7,8].

Only a few cases of CLN1 have been reported in Japan. Here, we report two cases of siblings with CLN1 juvenile onset type associated with type II diabetes mellitus. We also present the clinical, biochemical, and molecular characteristics of these cases.

## 2 Case presentation

**Fig. 1:**
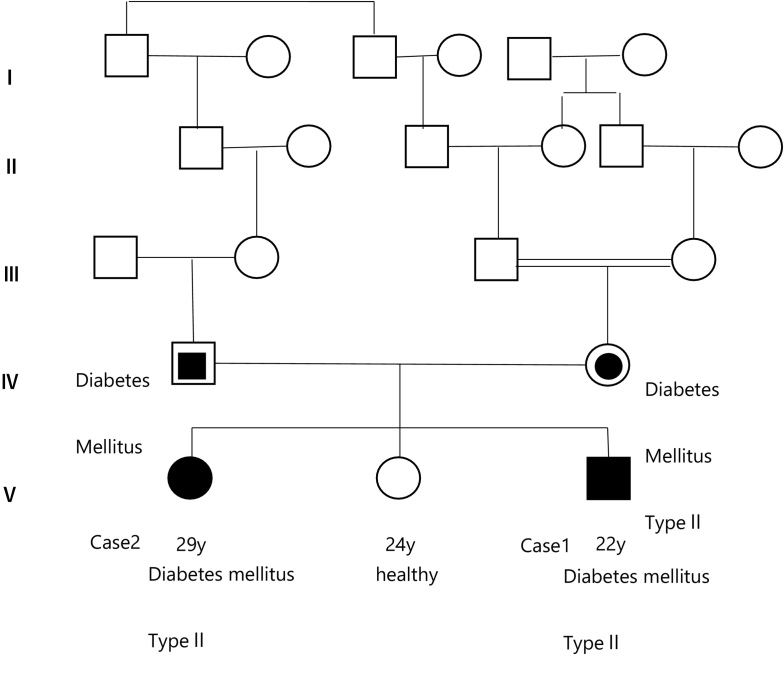
Genealogy of the cases. The patients' maternal grandparents were married cousins, and their paternal and maternal great-great-grandparents were brothers.

**Fig. 2:**
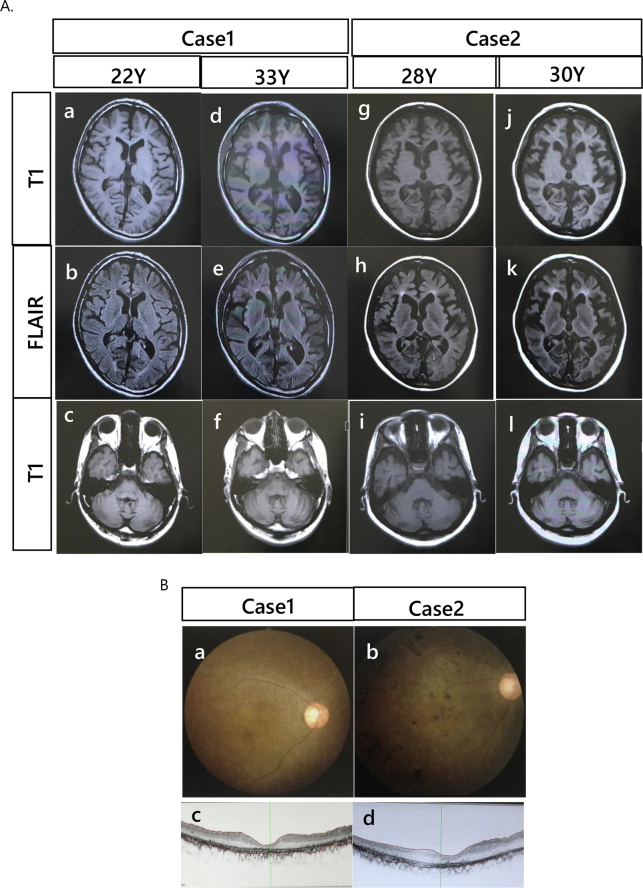
A. Serial changes in brain MRI in Case 1 (a–c: 22 years old, d–f: 33 years old) and Case 2 (g–i: 28 years old, j–l: 30 years old). Brain MRI showed progressive atrophy of the cerebrum and cerebellum and a high-intensity area in the FLAIR spectrum of the deep white matter. B. The Ocular fundus revealed pigmentary degeneration of the retina in Case 1(a) and Case 2(b). C. Optical coherence tomography (OCT) showing marked thinning of the retina in Case 1(a) and Case 2(b).

Case 1: A 22-year-old man was the youngest brother of three siblings. His maternal grandparents were married cousins, and his maternal and paternal great-great-grandfathers were siblings. The patient's parents also had type II diabetes mellitus (Fig. 1). There was no perinatal history or early developmental milestones. He had difficulty learning at the age of 8 years. Subsequently, vision deterioration and loss occurred, and the patient became blind at 12 years of age, with retinal pigmentary degeneration at 14 years of age. No epilepsy or ataxia was observed. He was diagnosed with diabetes mellitus and oral antidiabetic drugs were administered at 18 years of age. His mother visited the hospital for further examination at the age of 22 years. On neurological examination, the light reflex was sluggish, and eye movement was normal. Deep tendon reflexes (DTR) were normal, and ataxia was not observed. Blood examination revealed lymphocyte vacuolization. Blood glucose (2 h after meal) 106 mg/dL, HOMA-R 1.86 (Normal: <1.60), Insulin 7.1μU/mL (Normal range:1.5–16.4), HbA1c 6.9 (Normal range: 4.6–6.2%), Anti-GAD Ab <0.5 U/mL, Anti- IA-2Ab <0.6 U/mL, Insulin Ab <0.4 U/mL, T-cho 163 mg/dL, TG 51 mg/dL. Brain magnetic resonance imaging (MRI) revealed mild brain atrophy, enlargement of the lateral ventricle, and periventricular white matter lesion (Fig. 2, A). An ocular fundus examination revealed pigmentary retinal degeneration (Fig. 2, B). Optical coherence tomography (OCT) showed marked retinal thinning (Fig. 2, C).

Case 2: A 29-year-old woman was the oldest sister of the patient in Case1. There was no perinatal history or early developmental milestones. She had difficulty learning and dementia since the age of 10 years, with retinal pigmentary degeneration at 14 years of age. She was diagnosed with diabetes mellitus in her teenage years, and antidiabetic drugs were administered. She experienced difficulty sleeping at approximately 20 years of age. Ataxia was observed at the age of 28 years. She had difficulty walking; therefore, she used a wheelchair. She had no epilepsy. Her mother visited the hospital for further examination at the age of 29 years. She can communicate through simple speech and likes singing. She could not be evaluated intellectually because of visual impairment. She was regularly medicated for sleep disturbance and diabetes mellitus. Neurological examination revealed no light reflexes. Spasticity and ataxia were observed. Blood examination revealed lymphocyte vacuolization. Blood glucose (2 h after meal) 239 mg/ dL, HOMA-R 22.01(Normal: <1.60), Insulin 37.3μU/mL (Normal range: 1.5–16.4), HbA1c 7.4 (Normal range: 4.3–5.8%, JDS), Anti-GAD Ab <0.3 U/mL, Anti- IA-2Ab <0.6 U/mL, Insulin Ab <0.4 U/mL, T-cho 233 mg/dL, TG 371 mg/dL. The EEG showed a low voltage, and there was no epileptic discharge. Brain MRI revealed brain atrophy, enlargement of the lateral ventricle, and periventricular white matter lesion (Fig. 2, A). An ocular fundus examination revealed pigmentary retinal degeneration (Fig. 2, B). OCT showed marked retinal thinning (Fig. 2, C).

**Fig. 3:**
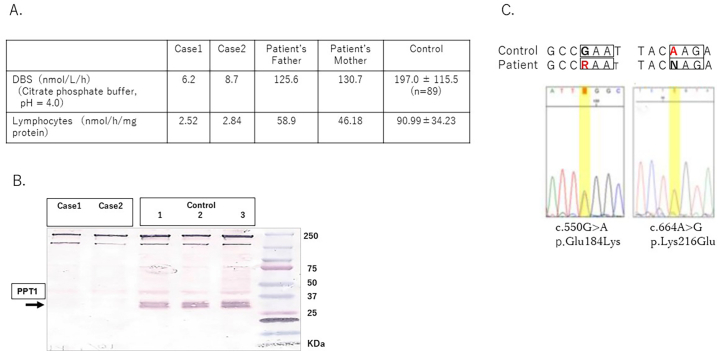
Biochemical and genetic findings of the patients. A. Measurement of PPT1 enzyme activity in DBS and Lymphocyte. B. PPT1 expression of the patients were decreased by Western blot analyses. C. *PPT1* gene analysis in the patients revealed compound heterozygote variants.

Both cases presented cognitive decline from school age and visual impairment occurred several years later. Therefore, NCL was suspected, particularly CLN3 or CLN1. The PPT1 activities of the patients and parents were measured using DBS and leukocytes/lymphocytes, according to a previously reported protocol [9]. Both of the patients showed reduced PPT1 enzyme levels, 6.2, 8.7 nmol/L/h in DBS and 2.52, 2.84 (nmol/h/mg protein), respectively. The PPT1 activity of the parents was also slightly reduced (Fig. 3, A). Western blot analysis also supported the reduced expression of PPT1 protein (Fig. 3,B). Genetic analysis of *PPT1* revealed compound heterozygous variants.

NM_000310.4(PPT1): c.550G > A and c.664 A > G[p.Glu184Lys and p.Lys216Glu](Fig. 3,C). The variants found in the patients were reported as the variants showing an infantile form in the former and a novel variant in the latter.

Twelve years have passed since the initial diagnosis. Patient 1 deteriorated gradually; he speaks words and moves with a chair while still walking for a few steps. There is no epilepsy, and electroencephalography (EEG) shows no obvious epileptic discharge. Brain MRI revealed progressive atrophy of the cerebrum and cerebellum. Patient 2 was placed in a wheelchair for mobility and had difficulty holding the trunk. She does not speak but can follow simple instructions. Seizures occurred at 33 years of age. EEG showed low background activity, and small spikes were dominant in the right frontal area. Sodium valproate, an antiepileptic drug, was started, and the seizures have since decreased. Nasogastric tube feeding was initiated at the age of 39 years to treat dysphagia.

## 3 Discussion

### 3.1 Clinical characteristics

Incidence of NCLs is estimated at 1.6–2.4/100,000 in US and at 2–7/100,000 in Europe. The exact estimated incidence of NCLs in Japan has not been calculated; however, a nationwide epidemiological survey in 2003 reported 27 patients. Few cases have been reported previously in CLN1: in Japan, two cases of infantile-onset have been reported, a 22-month-old boy with low PPT1 enzyme activity, and a 19-month-old girl with paternal uniparental isodisomy and juvenile onset type [[10], [11], [12]].

Our cases presented cognitive decline from school age, and visual impairment progressed several years later. NCL was suspected, and the presence of vacuoles in peripheral lymphocytes required differentiation from CLN3. Because visual impairment precedes intellectual regression in CLN3, we prioritized the enzymatic activity of CLN1 in these cases. A decrease in PPT1 activity was observed and genetic testing was performed to confirm the diagnosis. In the juvenile-onset form, CLN3 is suspected when lymphocyte vacuolization is present, but juvenile CLN1 is suspected when regressive symptoms precede visual impairment. If CLN1 enzyme activity is normal, genetic testing is recommended for the possibility of CLN3.

### 3.2 PPT1 enzyme deficiency and functional relation in neuronal cells

Compound heterozygous variants in *PPT1*, p.Glu184Lys and p.Lys216Glu which were observed in our cases, were predicted by POSSIBLY DAMAGING and PROBABLY DAMAGING using *PolyPhen-2* (p.Glu184Lys: Hum Div, 0.570 (sensitivity: 0.88; specificity: 0.91), p.Lys216Glu: Hum Div, 0.992 (sensitivity: 0.70; specificity: 0.97) in silico analysis.

The clinical features of p.Arg122Trp which is the most common homozygous variant in Finland, presents with infantile onset. This variant causes defects in the transport of gene products from the endoplasmic reticulum (ER) to the lysosomes. Analysis of the crystal structure of PPT1 predicted genotype–phenotype correlations in other variants [13,14]. The compound heterozygous variant p.Glu184Lys/p. Arg122Trp has been reported to exist in an infantile form. Glu184 is located at the palmitate-binding site of the protein. Substitution of Glutamic acid (Glu) with lysine (Lys) in the 184th amino acid caused a conformational change, according to crystal structure analysis [[13], [14], [15]]. The p.Lys216 is located in a potential N-glycosylation site, so substitution of this site may cause a structural change [14].

In addition, previous literature reports have shown that patients with low PPT1 activity (≤1% of baseline), including Japanese patients, tend to present with infantile-onset form, suggesting a correlation between PPT1 enzyme activity and the clinical presentation.

### 3.3 Relation to diabetes mellitus with PPT1 enzyme deficiency

The physiological function of PPT1 is a de-palmitoylating enzyme that releases various proteins (such as receptors, ion channels, and cell adhesion factors) that are bound to the cell membrane by palmitoylation from the plasma membrane. The accumulation of mutant PPT1 in the lipid raft fraction is thought to induce the excessive uptake of various proteins undergoing palmitoylation from the membrane into the cytoplasm, thereby inducing cellular damage.

Regarding complications of diabetes mellitus in patients with NCL, a case of CLN3 patient with insulin dependent diabetes mellitus has been reported previously [16], but not in CLN1. Thus, the correlation between the NCLs and diabetes mellitus remains unclear. Autoimmunity to glutamic acid decarboxylase (GAD) and alterations in mitochondrial function have been suspected in some types of NCLs.

#### 3.3.1 Autoimmunity and NCLs

GAD is an enzyme that functions in the production of gamma-aminobutyric acid (GABA) from glutamic acid, and anti-GAD antibodies are believed to inhibit GAD and suppress GABA production, resulting in neurological symptoms. Anti-GAD antibodies have been implicated in autoimmune encephalitis such as Stiff-Person syndrome (SPS), chronic cerebellar ataxia, and intractable epilepsy. The CLN3 mouse model shows a loss of GABAergic neurons, which are brain-associated antibodies to GAD that catalyze glutamic GABA. Anti-GAD antibodies have also been identified as autoantigens in autoimmune disorders type1 diabetes mellitus, although GAD autoantibodies in type I diabetes and SPS are known to differ in terms of epitope specificity, titer, and affinity. Even in CLN3, early studies suggest that the reactive epitope of GAD autoantibodies in CLN3 is distinct from them [17]. However, the involvement of GABA in CLN1 expression in the central nervous system has not yet been elucidated. Our patients tested negative for all autoantibodies, including anti-GAD antibodies, which are observed in type I diabetes mellitus, and we diagnosed type II diabetes mellitus based on the finding of insulin resistance.

#### 3.3.2 Mitochondrial abnormalities and NCLs

Acceleration of endocytosis through lipid rafts, abnormal autophagy, and mitochondrial dysfunction has been observed in cells derived from CLN1 patients. In the PPT1^−/−^ mouse model, PPT1^−/−^ neurons, compared to wild-type neurons, are more vulnerable to complex I inhibition by 1-methyl-4-phenylpyridinium (MPP), but less sensitive to complex IV inhibition by sodium azide, indicating that PPT1 deficiency causes alterations in the mitochondrial respiratory chain [18].

In fibroblasts from patients with infantile CLN1 disease, reduced activities of ATP synthase and respiratory chain complexes II, III, and IV have been reported [19]. Combined morphological and biochemical examinations revealed structural alterations in the mitochondrial compartment in the cytoplasm of CLN1 and CLN6 fibroblasts.

Using the intravital fluorescent marker Mito Tracker, a fragmented mitochondrial reticulum was observed in all cells, mainly in the perinuclear region. Accumulation of lysotracker- and Lysosome-associated membrane protein-2 (LAMP-2) positive lysosomal structures in the perinuclear region results in the distortion and fragmentation of the mitochondrial reticulum. Shifting to the periphery of JC-1 positive cells, which is an indicator of mitochondrial membrane potential, showed abnormal mitochondrial polarization and decreased expression of the mitochondria-related voltage channel protein VDAC-1 in CLN1 fibroblasts [20]. Thus, CLN1 may cause mechanical stress to the mitochondrial membrane and subsequent depolarization, followed by endoplasmic reticulum fragmentation and partial mitochondrial loss. There is no organ specificity in the distribution of the PPT1 protein and mRNA, and it is widely distributed in the brain and pancreas of humans [21] .

In our cases, although we cannot rule out the possibility that the complication of diabetes mellitus was coincidental, it is possible that mitochondrial dysfunction due to decreased PPT1 activity in the pancreas induced insulin resistance, leading to diabetes mellitus. However, a correlation between PPT1 deficiency and diabetes mellitus has not yet been established. Further investigation is required to clarify these precise mechanisms.

## CRediT authorship contribution statement

**Kaoru Eto:** Conceptualization, Data curation, Methodology, Investigation, Writing – original draft. **Rina Itagaki:** Data curation, Investigation, Visualization, Writing – review & editing. **Ayumi Takamura:** Data curation, Investigation, Visualization, Writing – review & editing. **Yoshikatsu Eto:** Writing – review & editing, Supervision. **Satoru Nagata:** Writing – review & editing, Supervision.

## Declaration of Competing Interest

The authors declare no conflicts of interest.

## Data Availability

The data that has been used is confidential.
